# Supplementary material for: Active Transport of Hepatotoxic Pyrrolizidine Alkaloids in HepaRG Cells
Source: Int J Mol Sci. 2021 Apr 7;22(8):3821. doi: 10.3390/ijms22083821 (PMC8067754; doi:10.3390/ijms22083821)
Supplement: Supplementary file 1 [file ijms-22-03821-s001.zip › Supplementary materials_Enge_proof read.docx]

- Supplementary Materials -

**Active transport of hepatotoxic pyrrolizidine alkaloids in HepaRG cells**

Anne-Margarethe Enge^1^, Florian Kaltner^2^, Christoph Gottschalk^2^, Albert Braeuning^1^, Stefanie Hessel-Pras^1^

ORCID-iDs: S. Hessel-Pras: 0000-0002-6153-0035

F. Kaltner: 0000-0003-2697-7461)

C. Gottschalk: 0000-0003-1292-8933)

A. Braeuning: 0000-0003-3810-027X)

^1^ German Federal Institute for Risk Assessment, Department of Food Safety, Max-Dohrn-Str.8-10, 10589 Berlin, Germany

^2^ Ludwig Maximilian University of Munich, Veterinary Faculty, Chair of Food Safety and Analytics, Schoenleutnerstr. 8, 85764 Oberschleissheim, Germany

Corresponding author:

Dr. Stefanie Hessel-Pras

German Federal Institute for Risk Assessment

Max-Dohrn-Str. 8-10, 10589 Berlin, Germany

E-mail: stefanie.hessel-pras@bfr.bund.de

Tel.: +49-30-18412-25203


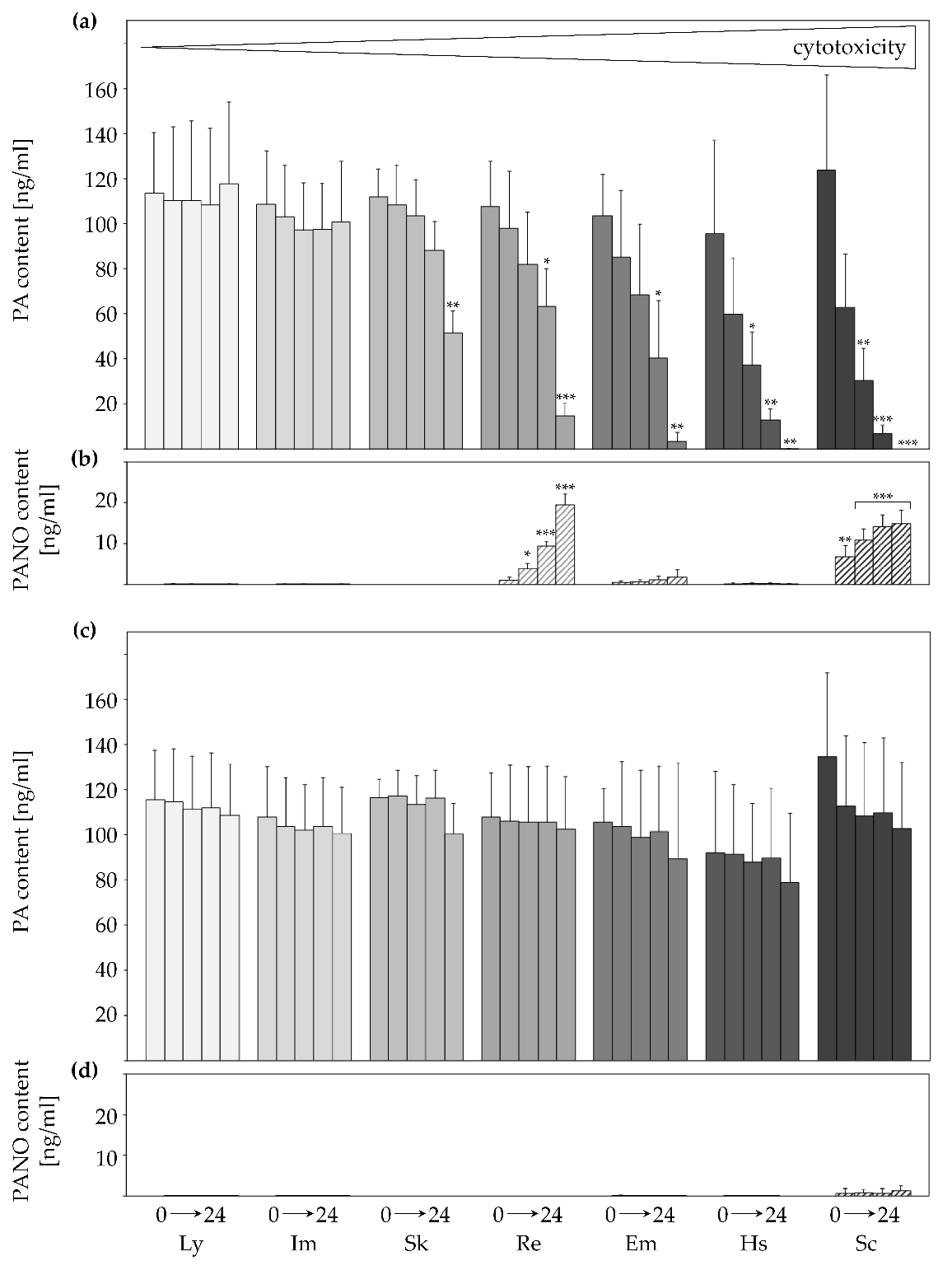


**Figure S1:** Time dependent reduction of seven structurally different PAs in cell culture medium of differentiated HepaRG cells under different temperatures of 37°C (**a,b**) and 4°C (**c,d**) in ng/ml. HepaRG cells were incubated for 2, 4, 8 and 24 h with 700 nM of lycopsamine, intermedine, senkirkine, retrorsine, echimidine, heliosupine or senecionine, respectively. Cell culture medium was collected after each time point and PA content (gray bars) and PANO content (striped bars) measured by LC-MS/MS. PA and PANO content is given in ng/ml as it was quantified in the 1:7 diluted samples as mean + SD of three to four independent experiments with two incubations each. Please note the unequal scaling of the different y-axes. Cytotoxicity grading was done using data from Glück et al. (2021) [41]. Statistical analysis was performed using one-way ANOVA, followed by Dunnett‘s post-hoc test, and significant differences are depicted as follows: * for PA and PANO content with *p < 0.05, ***p < 0.001. Ly, lycopsamine; Im, intermedine; Sk, senkirkine; Re, retrorsine; Em, echimidine; Hs, heliosupine; Sc, senecionine.


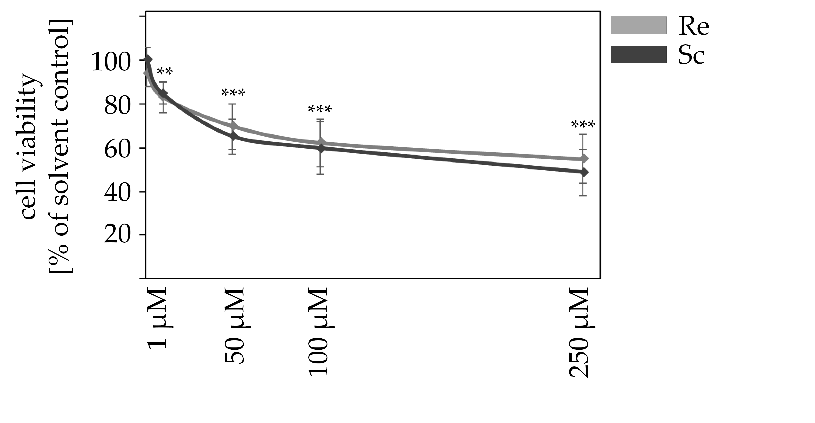


**Figure S2.** Effects of retrorsine and senecionine on cell viability of differentiated HepaRG cells. HepaRG cells were treated for 24 h with different concentrations (1 – 250 µM) of retrorsine or senecionine, respectively. Cell viability was assessed using MTT test as described in Section 4.4. Cell viability is displayed as percent of solvent control (2.5% ACN) as mean of six independent experiments with five different incubations each. 0.01% Triton X-100 was used as positive control, resulting in a decrease of cell viability below 0.5%. Statistical analysis was performed using one-way ANOVA, followed by Dunnett‘s post-hoc test, and significant differences are depicted as followed: **p < 0.01, ***p < 0.001. Re, retrorsine; Sc, senecionine.


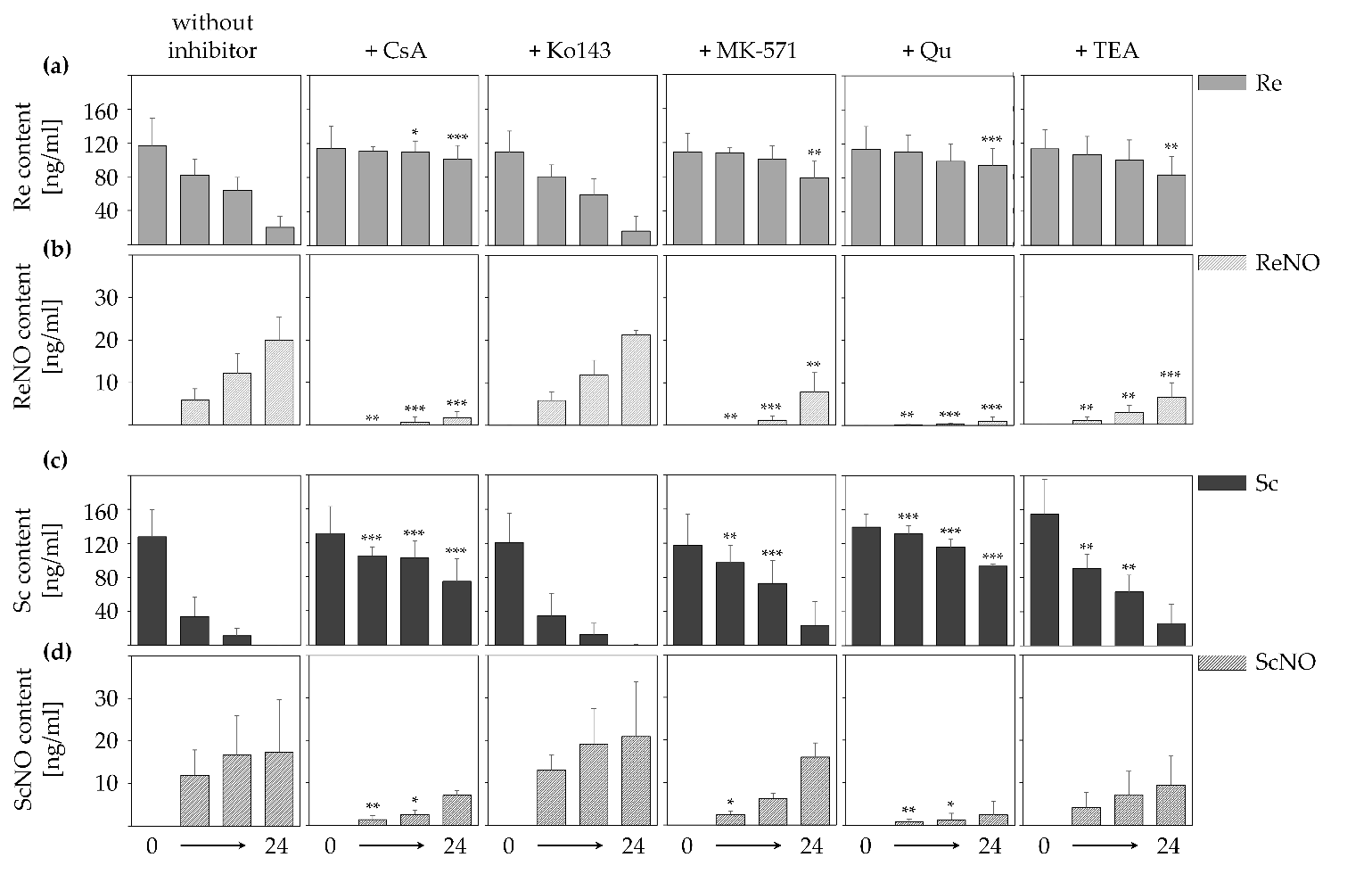


**Figure S3.** Effects of pharmacological influx and efflux inhibitors on time dependent reduction of retrorsine (**a,b**) and senecionine (**c,d**) in cell culture medium of differentiated HepaRG cells in ng/ml. HepaRG cells were pre-incubated for 1 h with the inhibitors CsA (10 µM), Ko143 (5 µM), MK-571 (50 µM), Qu (100 µM) and for 24 h with TEA (3 mM) before being incubated with 2.5 µM retrorsine or senecionine, respectively. Cell culture medium was collected after 4, 8 and 24 h and PA content (gray bars) and PANO content (striped bars) measured by LC-MS/MS. PA and PANO content is given in ng/ml as it was quantified in the 1:20 diluted samples as mean + SD of three to five independent experiments with two incubations each. Please note the unequal scaling of the different y-axes. Statistical analysis was performed using one-way ANOVA, followed by Dunnett‘s post-hoc test, and significant differences for each inhibitor in relation to the respective time point of the control group are depicted as follows: * for PA and PANO content at one time point for inhibitor-treatment vs. no-inhibitor with *p < 0.05, **p < 0.01, ***p < 0.001. Re, retrorsine; ReNO, retrorsine N-oxide; Sc, senecionine; ScNO, senecionine N-oxide.


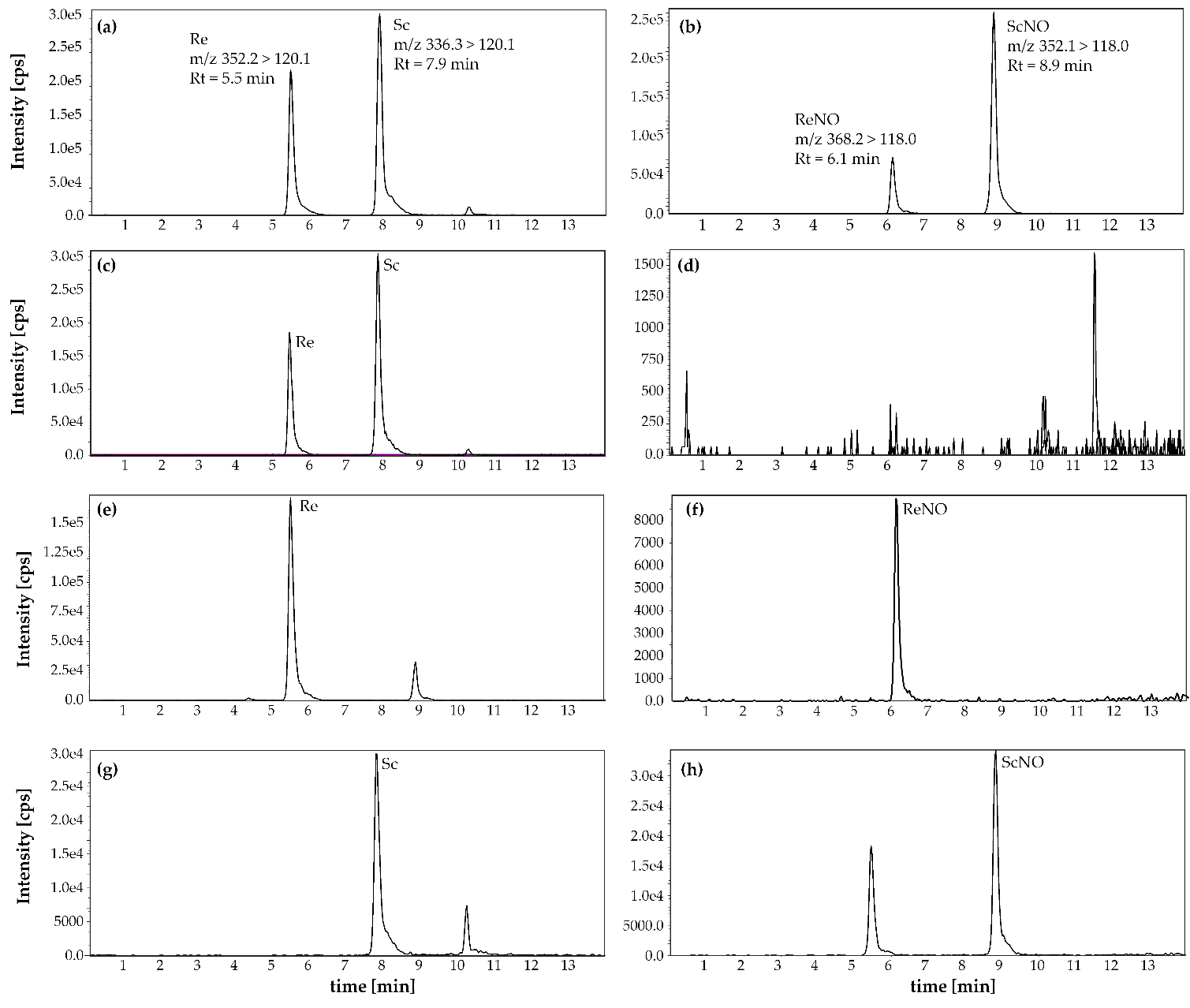


**Figure S4.** Representative chromatograms of LC-MS/MS analysis of senecionine, retrorsine and their *N*-oxides in cell supernatant of HepaRG cells treated with 2.5 µM senecionine or retrorsine, respectively. Prior to quantification, samples were diluted 1:20. For clarity, only one mass transition (quantifier) of each analyte is depicted: (a) 125 nM standard solution of senecionine (Sc) and retrorsine (Re), (b) 125 nM standard solution of senecionine *N*-oxide and retrorsine *N*-oxide (ScNO and ReNO, respectively), (c) Re and Sc at t_0_, (d) ReNO and ScNO at t_0_, (e) Re at t = 8 h, (f) ReNO at t = 8 h, (g) Sc at t = 8 h, and (h) ScNO at t = 8 h. Unlabeled peaks are non-specific signals of ion fragments of other substances contained in the sample. Rt = retention time; further LC-MS/MS parameters are summarized in Kaltner et al. (2019) [1].

**References of supplementary materials**

1. Kaltner, F.; Stiglbauer, B.; Rychlik, M.; Gareis, M.; Gottschalk, C. Development of a sensitive analytical method for determining 44 pyrrolizidine alkaloids in teas and herbal teas via LC-ESI-MS/MS. *Analytical and Bioanalytical Chemistry* **2019**, *411*, 7233-7249, doi:10.1007/s00216-019-02117-1.
